# The Didactic Method in Dental Teaching

**Published:** 1892-04

**Authors:** C. M. Wright

**Affiliations:** Cincinnati, Ohio, Prof. Physiology and General Pathology, Ohio College of Dental Surgery.


ARTICLE III.

### THE DIDACTIC METHOD IN DENTAL TEACHING.

BY C. M. WRIGHT, D. D. S., CINCINNATI, OHIO,

Prof. Physiology and General Pathology, Ohio College of Dental Surgery.

A cursory study of a great number of medical and dental periodicals for several years past develops the fact that medical, like the dental journals, have certain subjects which are continually coming to the surface in unexpected places. "Medical Education" and "Dental Education" are foremost among these pet subjects which are most frequently exploited.

Well-known writers, casual writers, editors, students, and others seem impelled by some impulse to write upon the subject of education at one time or another. In the large majority of these essays no greater harm is done than by the writers on the equally important subjects of "Success in life." It is quite possible that the same pens have pranced over the pages at one time to the tune of "Success," and to the tune of "Education" at another.

There may, however, be harm in the number of blows struck in ceaseless hammering away on anything, however intrinsically valuable it may be. Gold foil may be attenuated beyond its usefulness by too long-continued hammering. Crops of water constantly falling upon one spot will wear away the firmest substance, or shatter the most unflinching nerves. Essays upon education from any, every, and all sources, drop, drop, dropping in never-ending monotony upon the minds of teachers, make the didacticians tired, and in their weariness they may be induced to accept the doctrines of the persistent wordmakers on the subjects. In the hope of freeing themselves from a nuisance they may claim, "It is true! We accept (anything)! We agree! Now give us a rest!"

The particular doctrine which has been shot at the teachers in medical and dental schools from the pop-guns of epistolarians of all sorts for some years past, and which perhaps has worried them the most, is the one which affirms that "the lecture system is a failure." We may, indeed, now admit that this is a popular notion of the day. New colleges denounce the old method and boldly cry, "No lectures! All subjects taught by recitations! Hurrah!"

People read these flashing words, rub their hands, and say, "it is well; the lecture system must go."

Writers on education feel the tingling and jingling of the red blood-corpuscles in their peripheral capillaries, and smilingly admit that "this is the doctrine. *We* had to sit hour after hour and listen to old Professor A. and young Professor B. Did we learn anything? No. A thousand times No! Do we know anything now? Read the answer in—our essays on education!"

I do not wish to cast ridicule upon the *subject*. I do not mind, however, expressing myself forcibly against were writers,—writers who have not even learned the art of making their productions interesting. But when a practical man, a *teacher*, rises to explain I take off my hat with respect and listen attentively.

For years the methods in vogue and still generally employed in the dental as well as medical schools in teaching anatomy, physiology, chemistry, pathology, etc., were by lectures, illustrations, diagrams and experiments; the lectures supplemented by the so-called "quizzers," where a subject is pursued by questions on the part of the professor or quiz-master, and answers on the part of the student. In other words, by set oral examinations or recitations, consisting of questions and answers in reference to the various subjects embraced by the lectures. One or several good text-books are recommended, but the note-book has been the only recognized authority for the student. Thus much in explanation of the older method of instruction. Will anyone deny that for trained students this is an excellent method of teaching almost any branch of science? There are, however, certain faults associated with this method in classes made up of mixed elements. In fact, all classes must be made up of mixed elements. There are certain psychological and physiological reasons for this, which no one will dispute when he considers the various intellectual and physical capacities (varying in quality as well as in degree) which every class of men, even the smallest, must present.

Perhaps the most prominent mental faculty definitely localized in the cerebrum of the student, which the teacher in a professional or technical school must furnish and stimulate, is that which is known as memory. Just what impression goes to these gray cells in the brain of the student, just what condition the impression maintains in the centers, so that pictures, or sounds, or motile acts may be reproduced from the stored-up negative at future times, either automatically, from association, or at will, we do not know, but we have reasons to believe that perception is first in the order of mental and nervous operations,—perception of impressions received through special sense organs of various kinds—sight, hearing, touch, etc. The object of the text book and of the lecturer on any science or art is to awaken the perception,—to make vivid impression upon the perceptive faculties,—so that these impressions may effect the memory-cells. They can then be of use in the future. There is, as I have said, a difference in the susceptibility to impressions in the perceptive faculties of different minds.

This difference may be illustrated in inorganic substances by reference to the impressions of light. A sensitive plate used by a photographer, and a sheet of white paper, are both sensitive to light, and both can be made to impart images. The difference is in degree. This difference seems to exist to a marked degree in gray nervous matter, of which different individuals possess different qualities. In one we find a sensitive visual memory, in which pictures of objects that have been seen, as the printed page, the drawing, the figure, the thing represented by words, is *visible* to the mind, and can from it be reproduced. Another person has the auditive memory, and sounds, explanations by the voice of another, words heard in the lecture-room, form the clearest impressions stored away for future use in the memory's domain. It will not be necessary to go farther into this subject and refer to the so-called motile and concept memories. We can easily believe that if ten students sit down to a study of a chapter in a text-book, it may be safe to say that there will be ten impressions,—no two alike, either in character or intensity of shading. Some do not take impressions immediately or ever very distinctly from printed words. To be sure, with the chapter before them, repeated impressions can be made until the memory has been sufficiently impressed to assure the retention of the images. This is not true of the lecture unless the student is an expert note-taker, when by repetition he can secure the same advantage. Again, if these ten students sit down with their note-books and listen to a lecturer, who has learned by experience how to present his subject well, ten impressions will have been made, and we may expect a like difference in the distinctness, the shading, and the prominence of this or that portion that we found after the study of the text-book.

What can a teacher learn from these facts, if they are facts?

Can he become a partisan? Can he join the "Down-with-the-Lecture party? Please observe that there has been no dispute in regard to the value of *recitation* on the part of the student. Strictly speaking, there can be a combined *lecture and recitative* method, such as we have had for years, or a *text-book and recitative* method.

For a year past, in teaching physiology, I have confined myself to the text-book and recitative method exclusively. I wanted to test for myself the method that seems destined to become fashionable in law, medical and dental schools. I think I can say from my own experience that this method is a good one as far as it goes, but that it does not entirely meet the requirements of mixed classes, or the varying degrees of susceptibility to impressions necessarily present in all classes. Possibly it has no advantages over the lecture system, exclusively followed. From one year's experiment with the "no-lecture" plan, added to past experience of the old method (which, by the way, was the ladder by which we have all climbed to our present state of knowledge in medical, legal, scientific, and dental science, and which certain modern teachers and writers propose to ruthlessly kick aside), I am decidedly in favor of a combination of the good points in each method. I should recommend to the class a particular text-book on the subject to be taught, and should assign regular lessons. The students would be expected to come prepared to answer questions or to recite from the author on the lesson assigned. At another meeting, or at regular intervals during a session, the teacher should deliver lectures on the same topics, reviewing, explaining, illustrating the subjects, so that they would become illuminated. The interest of all of the students would be increased. The advantages of repetition would be gained. The student who has the auditive memory more keenly set to receive impressions, as well as the student with the more sensitive visual memory, would be benefitted by the stimuli to his different organs of perception. The habit of taking notes would be acquired, and in final reviews or in competitive examinations the note-book as well as the text-book would become an authoritative source of knowledge. All of these advantages to the student, it seems to me, would naturally follow the employment of the combination of lecture and text-book method as portrayed.

We have, I think, physiological, psychological and practical reasons for the adoption of the plan proposed.—*Dent. Cosmos.*

